# KPC-2-NDM-1-producing Serratia marcescens: first description in Peru

**DOI:** 10.1016/j.nmni.2022.101051

**Published:** 2022-11-18

**Authors:** Barbara Ymaña, Nestor Luque, Maria J. Pons, Joaquim Ruiz

**Affiliations:** 1 Grupo de Investigación en Dinámicas y Epidemiología de la Resistencia a Antimicrobianos - “One Health”, Universidad Científica del Sur, Lima, Peru; 2 Escuela de Medicina Humana, Facultad de Ciencias de la Salud, Universidad Peruana Union (UPeU), Lima, Peru

**Keywords:** Americas, Bacterial infection

## Abstract

Two clonal extensively-resistant *S. marcescens* were isolated in Peru. The presence and transferability of extended-spectrum β-lactamases and IMP, KPC, NDM, OXA-48 and VIM were determined. The concomitant presence of KPC-2 and NDM-1 was confirmed in both isolates, with KPC-2 being able to be transferred.

*Serratia marcescens* is a human pathogen that often exhibits high levels of resistance to antimicrobial agents, including carbapenems [1]. Carbapenemase-producing microorganisms are of special concern, especially when they co-produce more than one carbapenemase [1]. KPC and NDM rank among the most relevant and frequent carbapenemases, with KPC being considered the most frequent worldwide [1].

The present study aimed to describe and characterise the first *bla*_KPC-2_-*bla*_NDM-1_-carrying *S. marcescens* isolated in Peru.

Two non-causing disease *S. marcescens* were isolated from nasopharyngeal (isolate 5Q) and hand swabs (isolate 5P) from a patient at ICU admission. Antimicrobial susceptibility to 15 antimicrobial agents was established by disk diffusion and clonal relationships by repetitive extragenic palindromic polymerase chain reaction (REP-PCR) [2]. The presence of ESBLs was established phenotypically [2], while *bla*_IMP_, *bla*_KPC_, *bla*_NDM_, *bla*_OXA-48_ and *bla*_VIM_ were detected by PCR [3]. The entire *bla*_KPC_ and *bla*_NDM_ were amplified and sequenced (Macrogen, Seoul, South Korea) and their conjugability was established [2,4,5]. After each conjugation assay, 6 transconjugants were randomly selected and the presence of carbapenemases was determined by PCR. Co-transfer of resistance was established in one transconjugant representative of each assay.

The isolates displayed identical REP-PCR patterns, and were extensively resistant (XDR). Both isolates possessed the *bla*_KPC-2_ and *bla*_NDM-1_ genes, with only *bla*_KPC-2_ being conjugative. The transconjugants exhibited susceptibility to all tested antimicrobial agents except cephalosporins, carbapenems, as well as β-lactam plus β-lactamase inhibitors (Table 1).

**Table 1:** Characteristics of the microorganisms

	Strain	AMC	PTZ	Caz	Cro	Fep	Imp	Mem	Nal	Cip	Oflo	Ak	Gm	Nit	Sxt	ESBL	KPC	NDM
*S. marcescens*	5Q	R (6)^a^	R (11)	R (6)	R (6)	R (6)	R (15)	R (17)	R (6)	R (6)	R (6)	S (19)	R (6)	R (6)	R (6)	—	+	+
*S. marcescens*	5P	R (6)	R (10)	R (6)	R (6)	R (6)	R (18)	R (18)	R (6	R (6)	R (6)	S (18)	R (6)	R (8)	R (6)	—	+	+
*E. coli*	MC1061	S (23)	S (30)	S (30)	S (34)	S (27)	S (33)	S (40)	S (22)	S (48)	ND	ND	S (24)	S (25)	S (30)	ND	—	—
*E. coli*	TC1Q_1_^b^	R (10)	R (12)	R (18)	R (15)	R (16)	R (16)	I (20)	S (25)	S (40)	ND	ND	S (30)	S (24)	S (32)	ND	+	—
*E. coli*	TC6Q_2_^b^	R (10)	R (13)	I (20)	R (16)	R (20)	I (20)	I (21)	S (27)	S (46)	ND	ND	S (20)	S (30)	S (40)	ND	+	—

AMC: amoxicillin plus clavulanic acid; PTZ: piperacillin plus tazobactam; Caz: ceftazidime; Cro: cefotaxime; Fep: cefepime; Imp: imipenem; Mem: meropenem; Nal: nalidixic acid; Cip: ciprofloxacin; Oflo: ofloxacin; Ak: amikacin; Gm: gentamicin; ESBL: extended-spectrum β-lactamases; R: resistant; S: susceptible; ND: not determined.

All data were interpreted in following the European Committee on Antimicrobial Susceptibility Testing (EUCAST) guidelines (https://www.eucast.org/fileadmin/src/media/PDFs/EUCAST_files/Breakpoint_tables/v_11.0_Breakpoint_Tables.pdf), with the exception of nalidixic acid, which was analysed following the 2021 guidelines of the Clinical and Laboratory Standard Institute. *Escherichia coli* ATCC 25922 was used as quality control.

a Diameter halo (mm).

b As both isolates have identical REP-PCR profiles, resistance pattern and the same carbapenemases, the transferability study was done using the isolate 5Q as the donor strain.

The isolation of carbapenem-resistant *S. marcescens* is increasing worldwide [6], thus limiting the treatment options for this pathogen. In the present isolates, resistance to carbapenems was related to the presence of *bla*_KPC-2_ and *bla*_NDM-1_. The concomitant presence of *bla*_KPC_ and *bla*_NDM_ has been previously reported in *S. marcescens*, including South American countries such as Brazil [6].

Regarding Peru, a study reporting the isolation of *S. marcescens* carrying *bla*_KPC_ and *bla*_NDM_ has been found, with the microorganism being isolated from a physician cell-phone, but no data of exact KPC or NDM variant was reported [3]. To the best of our knowledge, this is the first report of an human *bla*_KPC-2_-*bla*_NDM-1_-carrying *S. marcescens* in Peru. These findings shows the hidden stable presence of *bla*_KPC-2_-*bla*_NDM-1_-carrying *S. marcescens* in the hospital and probably reflects the lack of reports of circulating isolates, rather than the rarity of this genotype in the area.

The silent introduction of determinants of resistance to antimicrobial agents in areas such as ICUs is of special concern, since they may be carried by potential pathogens, as in the present case, or be horizontal transferred to nosocomial pathogens, for instance by conjugation as in the present study. In a middle-income country, such as Peru, with uncontrolled access to antibacterial agents, and high levels of antimicrobial resistance in the community [2], this is probably a frequent, albeit little described, phenomenon.

The present report shows the presence of a non-disease causing XDR *S. marcescens* carrying *bla*_KPC-2_ and *blae*_NDM-1_ silently introduced in the ICU highlighting the need to implement surveillance measures in hospitals for early detection of non-disease causing microorganisms possessing antimicrobial resistant determinants of special concern.

## Declaration of competing interest

The authors declared no conflicts of interest.
